# Contributions of modifiable risk factors to increased dementia risk in depression

**DOI:** 10.1017/S0033291722003968

**Published:** 2023-10

**Authors:** Anouk F. J. Geraets, Anja K. Leist, Kay Deckers, Frans R. J. Verhey, Miranda T. Schram, Sebastian Köhler

**Affiliations:** 1 Alzheimer Centre Limburg, Maastricht University Medical Centre+ (MUMC+), Maastricht, the Netherlands;; 2 Department of Psychiatry and Neuropsychology, Maastricht University Medical Centre+ (MUMC+), Maastricht, the Netherlands; 3 Department of Internal Medicine, Maastricht University Medical Centre+ (MUMC+), Maastricht, the Netherlands; 4 School for Mental Health and Neuroscience (MHeNs), Maastricht University, Maastricht, the Netherlands; 5 School for Cardiovascular Diseases (CARIM), Maastricht University, Maastricht, the Netherlands; 6 Department of Social Sciences, University of Luxembourg, Esch-Sur-Alzette, Luxembourg; 7 Heart and Vascular Centre, Maastricht University Medical Centre+ (MUMC+), Maastricht, the Netherlands

**Keywords:** ageing, cohort, dementia, depression, epidemiology, lifestyle, mediation, prevention, risk factors

## Abstract

**Background:**

Individuals with depression have an increased dementia risk, which might be due to modifiable risk factors for dementia. This study investigated the extent to which the increased risk for dementia in depression is explained by modifiable dementia risk factors.

**Methods:**

We used data from the English Longitudinal Study of Ageing (2008–2009 to 2018–2019), a prospective cohort study. A total of 7460 individuals were included [mean(standard deviation) age, 65.7 ± 9.4 years; 3915(54.7%) were women]. Depressive symptoms were assessed with the Center for Epidemiologic Studies Depression Scale-8 (score ≥3) or self-reported doctor's diagnosis. Ten modifiable risk factors for dementia were combined in the ‘LIfestyle for BRAin health’ (LIBRA) score. Dementia was determined by physician diagnosis, self-reported Alzheimer's disease or the shortened version of the Informant Questionnaire on Cognitive Decline in the Elderly (average score ≥3.38). Structural equation modelling was used to test mediation of LIBRA score.

**Results:**

During 61 311 person-years, 306 individuals (4.1%) developed dementia. Participants aged 50–70 years with depressive symptoms had higher LIBRA scores [difference(s.e.) = 1.15(0.10)] and a 3.59 times increased dementia risk [HR(95% CI) = 3.59(2.20–5.84)], adjusted for age, sex, education, wealth and clustering at the household level. In total, 10.4% of the dementia risk was mediated by differences in LIBRA score [indirect effect: HR = 1.14(1.03–1.26)], while 89.6% was attributed to a direct effect of depressive symptoms on dementia risk [direct effect: HR = 3.14(2.20–5.84)].

**Conclusions:**

Modifiable dementia risk factors can be important targets for the prevention of dementia in individuals with depressive symptoms during midlife. Yet, effect sizes are small and other aetiological pathways likely exist.

## Introduction

The relationship between depression and dementia is complex and its aetiology is still not well understood (Bennett & Thomas, 2014). Depression is considered a major risk factor for dementia, with meta-analyses showing a two times higher risk on average (Diniz, Butters, Albert, Dew, & Reynolds, 2013; Ownby, Crocco, Acevedo, John, & Loewenstein, 2006). Comorbid depression in dementia is associated with a reduced quality of life (Shin, Carter, Masterman, Fairbanks, & Cummings, 2005), impaired activities of daily living (Park, Jun, & Park, 2014), physically aggressive behaviour (Lyketsos et al., 1999), greater health care utilization (Kunik et al., 2003), early institutionalization (Kales, Chen, Blow, Welsh, & Mellow, 2005) and higher mortality rates (Suh, Kil Yeon, Shah, & Lee, 2005). Furthermore, the effectiveness of antidepressant pharmacotherapies on depression in patients with comorbid depression and dementia remains questionable (Leyhe et al., 2017). Thus far, the pathophysiology underlying these two common brain diseases remains unclear, but cardiometabolic and lifestyle risk factors have been shown to increase the risk for both depression (Penninx, 2017) and dementia (Deckers et al., 2015).

Mounting evidence suggests that modifiable cardiometabolic and lifestyle factors are the key to dementia risk reduction (Livingston et al., 2017; Lourida et al., 2019), while studies suggest that individuals with depression have a higher propensity for poor lifestyle and lifestyle-related diseases that are themselves risk factors for dementia. For instance, adults with major depression have a less healthy diet (Payne, Hybels, Bales, & Steffens, 2006), engage less in physical activity and show higher levels of sedentary behaviour (Schuch et al., 2017). Other cardiometabolic and lifestyle-related risk factors for dementia include, e.g. hypertension, obesity, diabetes, cardiovascular disease and smoking (Deckers et al., 2015). All these risk factors are elevated in depression (Penninx, 2017) and might contribute to increased dementia risk in depression (Köhler & Thomas, 2018). However, whether cardiometabolic and lifestyle factors explain the increased risk of dementia in individuals with depression has not been studied.

Therefore, this study aims to investigate the extent to which the increased risk for dementia in individuals with symptoms of depression is explained by modifiable dementia risk factors. Previous research has found age, sex and socioeconomic differences in both depression (Labaka, Goñi-Balentziaga, Lebeña, & Pérez-Tejada, 2018; Lorant et al., 2003) and dementia (Jorm & Jolley, 1998; Montero-Odasso, Ismail, & Livingston, 2020). Therefore, we assessed whether the associations differed for age, sex, educational level and wealth, with the latter as proxy for socioeconomic status.

## Methods

### Study population and design

**Fig. 1.**
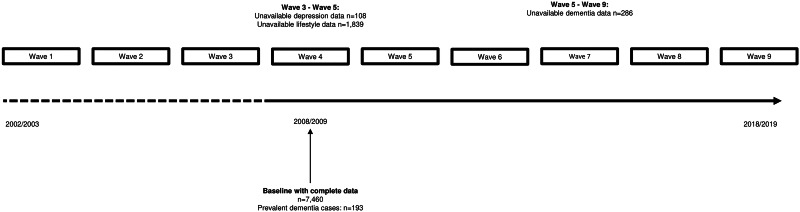
Study design.

Longitudinal data from the English Longitudinal Study of Ageing (ELSA) was used for this study. ELSA is a multi-centre panel study representing the English population aged 50 years and over (Steptoe, Breeze, Banks, & Nazroo, 2013). Measures of health, economics, psychology, lifestyle and social connections have been collected over bi-annual waves (Fig. 1). More details have been described previously (Rogers, Banks, Nazroo, & Steptoe, 2017).

Since wave 4 (2008/2009; *n* = 9886) includes a large number of modifiable risk factors for dementia, it was considered the baseline for this study. In case of missing information on depressive symptoms, risk and protective factors for dementia or potential confounders, non-missing data from adjacent waves [in most cases wave 3 (2006/2007) or wave 5 (2010/2011)] were used (online Supplementary eTable 1). The last follow-up measurement was wave 9 (2018/2019), yielding a maximum follow-up period of 11 years. In our analytical sample, participants with dementia at wave 4 (*n* = 193) and persons with missing depressive symptoms (*n* = 108) and lifestyle (*n* = 1839) data or loss to follow-up between waves 4 and 9 (e.g. withdrawal, death; *n* = 286) were excluded, resulting in a study sample of 7460 participants.

The National Health Service Multicenter Research and Ethics Committee and the University College London Research Ethics Committee approved the study according to the guidelines of the Helsinki Declaration. All participants provided written informed consent.

### Depressive symptoms

Depressive symptoms were assessed by the eight-item version of the Center for Epidemiologic Studies Depression Scale (CES-D 8) (Radloff, 1977). The CES-D 8 is an abbreviated version of the CES-D 20 (Turvey, Wallace, & Herzog, 1999) and commonly used in large population-based studies. The revised CES-D 8 contains a yes/no format for each question. To emphasize current state, participants were asked whether they experienced the symptom ‘much of the time during the past week’. A total CES-D 8 score ≥3 was used to denote ‘depressive symptoms’; this definition has been validated against standardized psychiatric interviews in older populations (Turvey et al., 1999). When one or two items were missing, depressive symptoms were scored as absent in case of a CES-D 8 score of 0 (1–2 missing) or CES-D 8 score of 1 (1 missing). Depressive symptoms were scored as missing in case of a CES-D 8 score of 1 and 2 missing items or more than 2 missing items. In addition, participants were assumed to have depressive symptoms in case of a self-reported doctor's diagnosis of depression.

### Dementia risk factors

Individual exposure to modifiable dementia risk factors was calculated using the LIfestyle for Brain Health (LIBRA) index (Deckers et al., 2015). This index includes diabetes, chronic kidney disease, coronary heart disease, cognitive and social activity, depression, healthy diet, hypertension, obesity, smoking, hypercholesterolemia, physical inactivity and alcohol use. Factors are dichotomized in ELSA according to pre-established cut-offs (Deckers et al., 2019a), and a weight is assigned to each factor, based on the factor's relative risk (Deckers et al., 2015). Weights are then standardized and summed to yield the final LIBRA score, indicating potential for dementia risk reduction, with a theoretical range of −5.9 to 12.7. Higher scores have been shown to predict cognitive decline and higher dementia risk in ELSA and other (population- and patient-based) cohort studies (Deckers et al., 2019b, 2020; Pons et al., 2018; Schiepers et al., 2018; Vos et al., 2017).

In ELSA, information was available for all LIBRA factors at wave 4, except for chronic kidney disease, based on clinical data from nurse visits or self-reported information. Because the determinant in this study concerns depressive symptoms, depression was omitted from the LIBRA score, resulting in a modified LIBRA score with a theoretical range from −5.9 to 9.5 (online Supplementary eTable 2).

### Dementia

Incidence of dementia was assessed at each wave by use of a combined algorithm of (1) self-reported or informant-reported physician-diagnosis of dementia or Alzheimer's disease; or (2) an average score of ≥3.38 on the 16-question Informant Questionnaire on Cognitive Decline in the Elderly (IQCODE) (Jorm, 1994, 2004; Jorm & Jacomb, 1989; Quinn et al., 2021). This questionnaire uses informant reports to evaluate the changes in every day cognitive function (e.g. memory) since the last interview (Jorm & Korten, 1988). Each item was scored on a 1 (much improved) to 5 (much worse) range. The validity of this scale was previously examined, and the threshold used has both high specificity (0.84) and sensitivity (0.82) (Jorm et al., 2004; Quinn et al., 2021).

### Confounders

Information on age, gender, educational level and wealth were collected through questionnaires. Educational level was regrouped into three categories: low (no formal qualifications), medium [ordinary (O-) level or secondary education (equivalent to high school)] or high [advanced (A-) level or above (college/university)]. Self-reported household wealth was calculated by summing wealth from the total value of a respondent's home (minus outstanding mortgage payments), physical wealth (e.g. jewellery), business assets (e.g. investments) and financial assets such as cash and savings (minus debts and loans). The overall measure of wealth was divided into tertiles.

### Statistical analyses

Analyses were conducted in Stata 14.1 (StataCorp, College Station, TX, USA) and Mplus 8 (Muthén & Muthén). Differences in demographics, depression, and LIBRA factors between individuals with and without incident dementia were evaluated using independent *t* tests, Mann–Whitney *U* tests or χ^2^ tests. Structural equation modelling for continuous-time survival analysis (Cox proportional hazard regression) was used to examine the associations between depressive symptoms, LIBRA and time to dementia, resulting in hazard ratios (HR) and their 95% confidence interval (CI). To study mediation by modifiable dementia risk factors on the association between depressive symptoms and incident dementia, the total effect of depressive symptoms was decomposed into direct and indirect effects (Pratschke et al., 2016). In all analyses, dementia was treated as the failure event. Age was used as the time axis, with survival time starting from age at wave 4 to age at dementia diagnosis (as reported by the participant or calculated as the mid-point between waves) or study exit (age of death or age at the last interview, whichever came first). Since participants could come from the same household, we used the Huber-White sandwich estimator to allow clustering at the household level (Williams, 2000). In addition, a sampling weight (baseline cross-sectional weight) was used in order to back-weight estimates from the analysis sample to the total sample to minimize selection bias. All analyses were adjusted for age, sex, educational level and wealth. We tested for interactions of depressive symptoms with age, sex, educational level and wealth on dementia risk. To assess whether the association of LIBRA score with dementia risk differed between participants with and without depressive symptoms, we tested the interaction of depression with LIBRA score on dementia risk. We tested mediation of the individual LIBRA factors in separate models to assess whether specific factors drive any of the mediation effects.

Several additional analyses were performed. First, to lower the risk of reversed causality, i.e. where dementia risk factors are consequences of the preclinical phase of dementia instead of risk factors (Solomon et al., 2014), we restricted analyses to those with 6 or more years of follow-up. Second, to reduce potential misclassification of participants with subthreshold depression (no diagnosis or low CES-D 8 scores due to remission or treatment), we adjusted for anti-depressant medication use and/or received counselling in the last 2 years. These data were only available in the subpopulation with a self-reported depression diagnosis, who had a high risk of confounding by indication and over-adjustment bias. Third, we performed a sensitivity analysis in which participants with one or two missing items on the CES-D were classified as missing. Because we imputed missing depression and LIBRA-score data at wave 4 with data from other waves, in sensitivity analyses we restricted assessment of depressive symptoms and behavioural data based on wave 4. Fourth, multiple imputation was used for missing values for depressive symptoms, the LIBRA factors, educational level and wealth. To impute missing values for depressive symptoms and the LIBRA factors, we used multivariate imputation by chained equations using all non-missing data on depressive symptoms/LIBRA factors, age and sex. For educational level and wealth status, we used the imputed depressive symptoms and LIBRA scores, age and sex (White, Royston, & Wood, 2011). Ten imputed datasets were created, and the results were combined using Rubin's rules (Rubin, 1996). A two-sided *p* value <0.05 was considered statistically significant.

## Results

### Baseline characteristics

During 61 311 person-years of follow-up, 306 participants (4.1%) developed dementia (*n* = 274 based on self-reported diagnosis and *n* = 32 additional based on the IQCODE), which yielded an incidence rate of 50 cases per 10 000 person-years. Table 1 shows the characteristics of the study sample at baseline, stratified for incident dementia. Participants had a mean age of 65.7 ± 9.4 years and 54.9% were women. Participants with incident dementia were older, had lower education and wealth, more often had depressive symptoms and had a worse dementia risk profile based on LIBRA compared to participants who did not develop dementia.

**Table 1.** Baseline characteristics of study sample

	Incident dementia		
	No (*n* = 7154)	Yes (*n* = 306)	*p* value
Demographics			
Age, mean (s.d.)	65.3 (9.2)	74.6 (8.4)	<0.001
Women, *n* (%)	3915 (54.7)	181 (59.2)	0.128
Educational level, *n* (%)			<0.001
Low	2813 (39.3)	161 (52.8)	
Medium	1952 (27.3)	76 (24.9)	
High	2386 (33.4)	68 (22.3)	
Wealth, *n* (%)			<0.001
Low	2209 (30.7)	120 (39.2)	
Medium	2458 (34.4)	107 (35.0)	
High	2487 (34.8)	79 (25.8)	
Depression			
CES-D 8 score ≥3, *n* (%)	1384 (19.5)	86 (28.4)	<0.001
Self-reported diagnosis^a^, *n* (%)	427 (6.0)	17 (5.6)	0.501
Combined, *n* (%)	1595 (22.3)	97 (31.7)	<0.001
Health- and lifestyle factors			
Heart disease, *n* (%)	692 (9.7)	59 (19.3)	<0.001
Diabetes, *n* (%)	1173 (16.4)	62 (20.3)	0.075
Hypercholesterolemia, *n* (%)	4474 (62.5)	180 (58.8)	0.189
Hypertension, *n* (%)	4258 (59.5)	226 (73.9)	<0.001
Obesity, *n* (%)	2332 (32.6)	89 (29.1)	0.199
Smoking, *n* (%)	943 (13.2)	32 (10.5)	0.166
Low-to-moderate alcohol use, *n* (%)	3975 (55.6)	135 (44.1)	<0.001
Physical inactivity, *n* (%)	1870 (26.1)	137 (44.8)	<0.001
High cognitive and social activity, *n* (%)	3023 (42.3)	79 (25.8)	<0.001
Healthy diet, *n* (%)	4273 (59.7)	163 (53.3)	0.024
LIBRA score, mean (s.d.)	0.22 (3.03)	1.40 (2.69)	<0.001

*n* = 7460. CES-D indicates Center for Epidemiologic Studies Depression Scale-8; LIBRA, LIfestyle for BRAin health, s.d., standard deviation. *p* values are presented for the comparison of individuals with and without dementia at follow-up (independent samples *t* tests, Mann–Whitney *U* tests and χ^2^ tests). LIBRA score theoretical range: −5.9 to 9.5; observed range: −5.9 to 9.5.

a Only assessed in subsample. Figures may not sum to 100% because of rounding errors.

v

Participants with incomplete data (*n* = 2233) were older, more often had depressive symptoms, had lower levels of education and wealth, more unfavourable health conditions (except hypercholesterolemia and obesity), a less healthy lifestyle and lower LIBRA scores compared to participants with complete data (online Supplementary eTable 3).

### Association of depressive symptoms with dementia

**Fig. 2.**
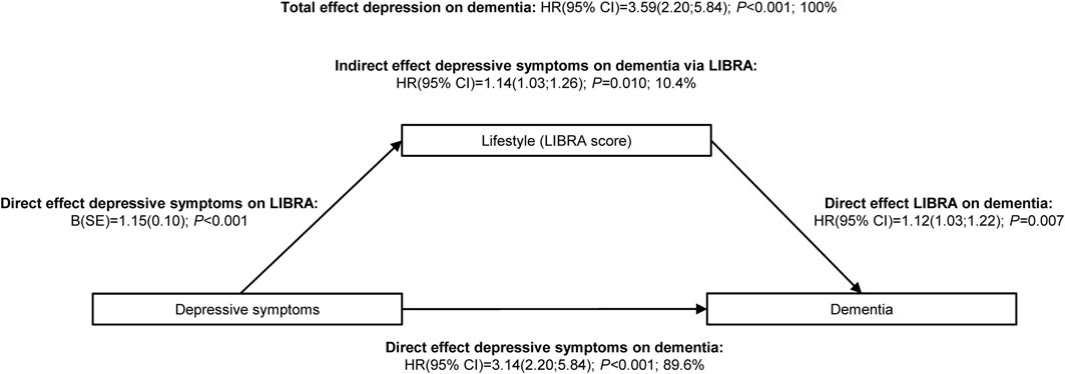
Decomposed associations of depressive symptoms with incident dementia in subpopulation aged 50–70 years. *n* = 5015; 77 dementia cases. LIBRA indicates LIfestyle for BRAin health; B, unstandardized regression coefficient; s.e., standard error; HR, hazard ratio; CI, confidence interval. Analyses are adjusted for age, sex, educational level, wealth and clustering at the household level.

The incidence rate of dementia was 73 and 43 per 10 000 person-years in participants with and without depressive symptoms, respectively. In the adjusted model, depressive symptoms were associated with increased risk for dementia [total effect; HR = 1.64(1.25–2.16); *p* < 0.001; online Supplementary eTable 4]. There was an interaction of depressive symptoms with age (*p* interaction < 0.002) on dementia risk: in younger individuals the association between depressive symptoms and dementia was stronger than in older individuals. To illustrate this we stratified analysis for age < and ≥70 years because the numbers of dementia cases within other age bounds (<60 and <65 years) were too small in the younger age categories to perform mediation analyses (10 and 34 cases, respectively), but the interaction applies to the complete age range: in participants aged <70 years, the association between depressive symptoms and dementia was stronger [HR = 3.59(2.20–5.84); *p* < 0.001; Fig. 2], while in participants aged ≥70 years the association attenuated and became non-significant [HR = 1.19(0.77–1.48); *p* = 0.297; online Supplementary eTable 4]. We did not find interactions of depressive symptoms with sex, educational level or wealth on dementia risk (online Supplementary eTable 5).

In the above results, we only found an association between depressive symptoms and dementia in participants aged 50–70 years. Therefore, further analyses are performed in the subpopulation aged <70 years. Mediation analyses in the total study population and the subpopulation aged ≥70 years are shown in online Supplementary eTable S4.

### Association of depressive symptoms with lifestyle and lifestyle with dementia

Depressive symptoms were associated with a higher LIBRA score [adjusted mean difference = 1.15, standard error (s.e.) = 0.10; *p* < 0.001; Fig. 2; subpopulation aged <70 years], indicating a worse lifestyle and a higher dementia risk. A one-point higher LIBRA score was, on average, associated with a 12% increase in dementia risk [HR = 1.12(1.03–1.22); *p* = 0.007; subpopulation aged <70 years].

### Mediation analysis

LIBRA significantly mediated the association between depressive symptoms and incident dementia in participants aged <70 years (Fig. 2). Mediation analysis showed an indirect effect of depressive symptoms on dementia risk via LIBRA [HR = 1.14(1.03–1.26); *p* = 0.010], in which 10.4% of the association between depressive symptoms and dementia risk was explained by LIBRA. The direct effect of depressive symptoms on dementia risk remained significant [HR = 3.14(2.20–5.84); *p* < 0.001] and explained the remaining 89.6% of the association between depressive symptoms and dementia risk.

There was no significant interaction of depressive symptoms with LIBRA on dementia risk (*p* interaction = 0.592; online Supplementary eTable 5), suggesting a similar relation of lifestyle with dementia risk for participants with and without depressive symptoms.

Of the individual LIBRA factors, only physical inactivity [HR = 2.27(1.39–3.71); *p* = 0.001] and cognitive and social activity [HR = 0.49(0.28–0.84); *p* = 0.010] were significantly associated with incident dementia. However, a continuous measurement to decompose the total effect into direct and indirect effects was only available for cognitive and social activity in ELSA. Therefore, further mediation analysis was only performed for cognitive and social activity (Table 2). There was an indirect effect of depressive symptoms on dementia risk via cognitive and social activity [HR = 1.12(1.04–1.21), *p* = 0.002], while the direct effect remained significant [HR = 3.11(1.89–5.13), *p* < 0.001].

**Table 2.** Decomposed associations of depressive symptoms with dementia by cognitive and social activity in subpopulation aged 50–70 years

	B	s.e.	HR	Lower CI	Upper CI	*p* value
Depressive symptoms on cognitive and social activity^a^	0.678	0.083	–	–	–	<0.001
Cognitive and social activity^a^ direct on dementia	–	–	0.84	0.76	0.94	0.001
Depressive symptoms direct on dementia	–	–	3.11	1.89	5.13	<0.001
Depressive symptoms indirect on dementia	–	–	1.12	1.04	1.21	0.002
Depressive symptoms total on dementia	–	–	3.49	2.15	5.69	<0.001

*n* = 5015; 77 dementia cases. B indicates unstandardized regression coefficient; s.e., standard error; HR, hazard ratio; CI, confidence interval. Analyses are adjusted for age, sex, educational level, wealth and clustering at the household level.

a Cognitive and social activity was entered as a continuous variable with a score range from 0 to 15.

### Additional analyses

Sensitivity analyses are shown in online Supplementary eTable 6. Analysis restricted to those with 6 or more years of follow-up to lower the risk for reversed causality, and adjustments to reduce potential misclassification of participants with subthreshold depression, did not change the results. Though no statistically significant interactions were found of depressive symptoms with sex, educational level and wealth, stratified analyses suggested stronger effects from depressive symptoms to dementia for men, participants with a medium or high educational level and for participants with a high wealth (online Supplementary eTable 7). Restriction of depressive symptoms and behaviour data to available wave 4 data did not change our results (data not shown). After multiple imputation, the total analysis sample consisted of 8933 participants, of whom 395 (4.4%) developed dementia (online Supplementary eTable 8). In line with the primary Cox proportional hazard regression analyses, a one-point higher LIBRA score was associated with a 9% increased dementia risk [HR = 1.09(1.04–1.14); *p* < 0.001] and depressive symptoms were associated with a 50% increased risk of dementia [HR = 1.50(1.18–1.90); *p* = 0.001].

## Discussion

In this population-based study, depressive symptoms were associated with poorer lifestyle and increased risk of dementia in midlife (age 50–70 years). The excessive risk for dementia was partly explained by a higher prevalence of lifestyle and health-related dementia risk factors in depressive symptoms. Our findings suggest that improving lifestyle in midlife in individuals with symptoms of depression may reduce the difference in dementia risk between individuals with and without symptoms of depression to some degree, while additional pathways explain the larger residual dementia risk in depression.

Our finding that depressive symptoms are associated with incident dementia corroborates previous evidence of a relation between depression and increased dementia risk (Diniz et al., 2013), and confirms studies that show that comorbid cognitive impairment is often persistent (Köhler, Thomas, Barnett, & O'Brien, 2010) and can increase over time (Mourao, Mansur, Malloy-Diniz, Castro Costa, & Diniz, 2016), with 45% of patients having persistent cognitive impairment even after remission of depression (Thomas & O'Brien, 2008). The observed association between depressive symptoms and a less healthy lifestyle has also been demonstrated in previous studies. Smoking, excessive alcohol use, physical inactivity, unhealthy diet, lower treatment compliance and worse medical care have been proposed as underlying mechanisms linking depression to cardiovascular health (Penninx, 2017). In a study of primary care patients, individuals with depression who developed incident hypertension or incident stroke were at particular risk of subsequent dementia (Köhler, Buntinx, Palmer, & van den Akker, 2015). The current study extends these observations by showing that a lifestyle-based dementia risk score explains 10.4% of the excessive dementia risk in individuals with symptoms of depression. Hence, while non-significant interaction analyses suggested that lifestyle was of equal importance to dementia risk in participants with and without depressive symptoms, participants with symptoms of depression had worse LIBRA scores on average, and so a larger proportion could probably benefit from lifestyle changes.

Additional analyses showed that physical activity and engagement in cognitive and social activities were the most important factors for the increased dementia risk among individuals with symptoms of depression. Excessive sedentary behaviour and disengagement in cognitive and social activities are related to core symptoms of depression. Physical inactivity is related to worse cardiometabolic outcomes, cerebral small vessel disease, higher inflammatory activity and lower brain-derived neurotrophic factor levels, which are all proposed as biological mechanisms underlying depression (Alexopoulos, 2019). Lower cognitive and social activity might be related to lower brain reserve as indicated by changes in structural and function brain connectivity (Kaiser, Andrews-Hanna, Wager, & Pizzagalli, 2015; van Velzen et al., 2020). A variety of lifestyle modifications have been developed to manage depression alongside pharmacological and psychological interventions (Sarris, O'Neil, Coulson, Schweitzer, & Berk, 2014), and our findings support integrative approaches for depression treatment, including lifestyle modification targeting sedentary behaviour and cognitive and social inactivity.

Though modifiable dementia risk factors included in LIBRA partly explained increased risk of dementia in depressive symptoms, a large proportion (89.6%) of this risk remained unexplained. Part of the residual risk might be due to other modifiable dementia risk factors such as sleep, hearing impairment or air pollution (Livingston et al., 2020). It is important to note that although observational evidence suggests that lifestyle interventions can reduce the risk of dementia, evidence from randomized controlled trials showed low or no effect of lifestyle intervention on dementia risk (Montero-Odasso et al., 2020). However, even relatively small reductions in dementia risk can have a major public health impact. Furthermore, lifestyle interventions may improve general health; they are relatively inexpensive and have minimum adverse effects. Therefore, large clinical trials that test the effectiveness of lifestyle intervention in diverse subpopulations with a long follow-up duration are recommended. Recent studies with extensive follow-up implied that depressive symptoms are likely a prodromal symptom of dementia (Mirza et al., 2016). While it is difficult to disentangle the role of depression as a risk factor or early symptom of dementia in late-life, the independent role of depression as risk factor at earlier as well as later life stages has been well established (Livingston et al., 2020). In addition, our findings suggest that aetiological heterogeneity exists in the complex relation between depressive symptoms and dementia. Notably, in ELSA, the association between depressive symptoms and incident dementia was stronger in participants aged 50–70 years, while the association attenuated and became non-significant in participants aged ≥70 and above (except of the lifestyle-mediated part of excessive risk). This is in line with a previous study which suggested that recurrent depression over the life course may be aetiologically associated with increased risk of vascular dementia, while late-onset depression is part of Alzheimer's disease (Barnes et al., 2012). Individuals might have recurrent depression, and lifestyle modification might especially be important for the prevention of vascular dementia in individuals with depressive symptoms in midlife. Stratified analyses suggested stronger associations between depressive symptoms and dementia risk in men, higher-educated and wealthy participants. Further studies are needed to replicate our findings and to further investigate socioeconomic differences in the associations of depression, lifestyle, with dementia.

Strengths of our study include its large sample size and population-based design; an extended follow-up period with biennial assessments; the extensive assessment of modifiable dementia risk factors; and the performance of several sensitivity analyses to test the robustness of findings and provide insight into which modifiable dementia risk factors are the most important in depression.

This study also has some limitations. First, the longitudinal population-based design comes along with non-response, selection and attrition bias (Banks, Muriel, & Smith, 2011). This may have led to a healthier study sample and therefore associations may be underestimated. However, sampling weights were implemented to minimize selection bias, and we performed multiple imputation in sensitivity analyses, which resulted in similar result patterns. Second, dementia is still underdiagnosed (Lang et al., 2017). Incidence rates of dementia might therefore have been underestimated. Third, dementia diagnosis, depressive symptoms and some modifiable dementia risk factors were partly based on self-reported data. Consequently, depressive symptoms measured with a questionnaire are not equivalent to a clinical diagnosis of major depressive disorder. Fourth, modifiable risk factors for dementia may interact with each other, which is not considered in the LIBRA score. Fifth, we cannot rule out the risk of residual confounding. Unmeasured factors, like hearing impairment, might have influenced the increased risk for dementia in depressive symptoms. Lastly, there might have been reversed causality in which dementia risk factors are consequences of the preclinical phase of dementia instead of risk factors (Solomon et al., 2014). However, sensitivity analysis in which we excluded participants with less than 6 years of follow-up did not materially change our results.

In this population-based cohort study, depressive symptoms were associated with an increased risk for dementia in midlife (age 50–70 years). The increased dementia risk in depressive symptoms was partly explained by elevated cardiometabolic and lifestyle risk factors in individuals with depressive symptoms. Although modifiable dementia risk factors only modestly explained the increased risk for dementia in depressive symptoms, they are suggested to be an important target for the prevention of dementia in depression, as even a small reduction in dementia risk would have tremendous public health impact on population health.

## Supplementary material

For supplementary material accompanying this paper visit https://doi.org/10.1017/S0033291722003968. click here to view supplementary material
