# Supplementary material for: Contributions of modifiable risk factors to increased dementia risk in depression
Source: Psychol Med. 2023 Feb 3;53(14):6583–91. doi: 10.1017/S0033291722003968 (PMC10600930; doi:10.1017/S0033291722003968)
Supplement: Supplementary file 1 [file S0033291722003968sup001.docx]

**Supplementary Material**

**Contributions of Modifiable Risk Factors to Increased Dementia Risk in Depression**

Anouk F.J. Geraets, Ph.D.^1,2,3,5,6,7^, Anja K. Leist, Ph.D.^7^, Kay Deckers, Ph.D.^1,2,5^, Frans R.J. Verhey, M.D., Ph.D.^1,2,5^, Miranda T. Schram, Ph.D.^2,3,4,5,6^, Sebastian Köhler, Ph.D.^1,2,5^

^1^Alzheimer Centre Limburg, ^2^Department of Psychiatry and Neuropsychology, ^3^Department of Internal Medicine, ^4^Heart and Vascular Centre, Maastricht University Medical Centre+ (MUMC+), Maastricht, the Netherlands; ^5^School for Mental Health and Neuroscience (MHeNs), ^6^School for Cardiovascular Diseases (CARIM), Maastricht University, Maastricht, the Netherlands; ^7^Department of Social Sciences, University of Luxembourg, Esch-Sur-Alzette, Luxembourg

**eTable 1**. Strategies to reduce missing values (if information at ELSA Wave 4 was missing).

| **Variable** | **Strategies to reduce missing values** |
| --- | --- |
| Depressive symptoms | - Look at identical information from ELSA Wave 5 and Wave 3 |
| Diabetes | - Look at self-report at ELSA Wave 2 and 3 for ever reported diabetes - Look at self-report at ELSA Wave 5-9 for never reported diabetes |
| Heart disease | - Look at self-report ELSA Wave 1-4 for ever reported heart disease - Look at self-report ELSA Wave 5-9 for never reported heart disease |
| Physical inactivity | - Look at identical information from ELSA Wave 5 and Wave 3 |
| Smoking | - Look at identical information from ELSA Wave 5 and Wave 3 |
| Hypertension | - Look at self-report and medication use from ELSA Wave 5 and Wave 3 |
| Hypercholesterolemia | - Look at self-report and medication use from ELSA Wave 5 and Wave 3 |
| Low-to-moderate alcohol use | - Look at identical information from ELSA Wave 5 |
| High cognitive and social activity | - Look at identical information from ELSA Wave 5 and Wave 3 |
| Obesity | - Look at identical information from ELSA Wave 6 |
| Healthy diet | - Look at identical information from ELSA Wave 5 and Wave 3 |
| Wealth | - Look at identical information from ELSA Wave 3 and Wave 5 - Look at identical information from ELSA Waves 1,2,6,7 |
| Educational level | - Look at identical information from ELSA Wave 5-9 (when information from   Wave 4 and previous Waves was not available) |

**eTable 2**. LIBRA operationalization and weights in the English Longitudinal Study of Ageing

| **Factor** | **Operationalization** | **Weight** |
| --- | --- | --- |
| Heart disease | Physician’s diagnosis of angina pectoris or myocardial infarction. | +1.0 |
| Diabetes (type-2) | Self-reported diagnosis, blood glycated hemoglobin level ≥ 48 mmol/mol (6.5%) according to the WHO guidelines [1] or use of glucose lowering medication. | +1.3 |
| Hypercholesterolemia | Self-reported diagnosis, total cholesterol level of ≥5.0 mmol/L and low-density lipoprotein of ≥3.0 mmol/L, following the guidelines of the National Health Service UK [2] or use of lipid-modifying medication. | +1.4 |
| Hypertension | Self-reported diagnosis, mean systolic blood pressure ≥140 mm Hg or mean diastolic blood pressure ≥90 mm Hg [3] or use of blood pressure lowering medication. | +1.6 |
| Obesity | Established cut-offs according to the WHO guidelines [6]. Waist circumference (men: > 102 cm; women: > 88 cm) and waist-to-hip ratio (men: >90; women: >85) were only used if data on body mass index (BMI ≥ 30) was missing. | +1.6 |
| Smoking | Self-reported current smokers or non-smokers. | +1.5 |
| Low-to-moderate alcohol use | Self-reported frequency of any alcohol consumed in the past 12 months. Low-to-moderate alcohol use was defined as 1-14 glasses per week according to recent UK alcohol guidelines [7]. | -1.0 |
| Physical inactivity | Self-reported engagement in vigorous, moderate or mild physical activity during leisure time (more than once per week, once per week, one to three times per month, hardly ever). Participants were dichotomized into physically active (≥1/week) or physically inactive (1-3 times/month, hardly ever/never). | +1.1 |
| High cognitive and social activity | Self-reported engagement in intellectual and social activities undertaken in the last 12 months (e.g., read the newspaper on a daily basis, have a hobby, take a holiday, using the internet, being a member of any organizations, clubs or societies). Engagement in seven or more of these activities was considered as cognitively active (distribution-based cut-off). | -3.2 |
| Healthy diet | Reported amount of fruits and vegetables consumed by the participant the previous day. A healthy diet was defined as consuming five or more portions of fruits and vegetables on a daily basis [8]. | -1.7 |
|  |  |  |
| Total theoretical LIBRA range without depressive symptoms |  | -5.9 to +9.5 |

LIBRA indicates LIfestyle for BRAin health; UK, United Kingdom; WHO, World Health Organization.

**eTable 3.** Characteristics of individuals included and excluded in the study

|  | **Included (n=7,460)** | **Excluded (n=2,233)** | ***p-value*** |
| --- | --- | --- | --- |
| **Demographics** |  |  |  |
| Age, mean (SD) | 65.7 (9.4) | 67.4 (11.2) | **<0.001** |
| Women, n (%) | 4,096 (54.9) | 1,265 (56.7) | 0.146 |
| Educational level, n (%)  Low  Medium  High | 2,974 (39.9)  2,028 (27.2)  2,454 (32.9) | 1,172 (53.0)  501 (22.7)  538 (24.3) | **<0.001** |
| Wealth, n (%)  Low  Medium  High | 2,329 (31.2)  2,565 (34.4)  2,566 (34.4) | 922 (41.3)  711 (31.8)  600 (26.9) | **<0.001** |
| **Depression** |  |  |  |
| CES-D 8 score >=3, n (%) | 1,470 (19.8) | 578 (28.5) | **<0.001** |
| Self-reported diagnosis *, n (%) | 444 (6.0) | 135 (6.1) | 0.501 |
| Combined, n (%) | 1,692 (22.7) | 660 (31.1) | **<0.001** |
| **Health- and lifestyle factors** |  |  |  |
| Heart disease, n (%) | 751 (10.1) | 286 (12.8) | **<0.001** |
| Diabetes, n (%) | 1,235 (16.6) | 1,281 (57.4) | **<0.001** |
| Hypercholesterolemia, n (%) | 4,654 (62.4) | 724 (40.9) | **<0.001** |
| Hypertension, n (%) | 4,484 (60.1) | 1,739 (84.4) | **<0.001** |
| Obesity, n (%) | 2,421 (32.5) | 380 (34.3) | 0.222 |
| Smoking, n (%) | 975 (13.1) | 374 (17.1) | **<0.001** |
| Low-to-moderate alcohol use, n (%) | 4,110 (55.1) | 700 (48.8) | **<0.001** |
| Physical inactivity, n (%) | 2,007 (26.9) | 948 (42.5) | **<0.001** |
| High cognitive and social activity, n (%) | 3,102 (41.6) | 495 (24.7) | **<0.001** |
| Healthy diet, n (%) | 4,436 (59.5) | 948 (55.0) | **0.001** |
| LIBRA score, mean (SD) | 0.27 (3.02) | 1.82 (2.77) | **<0.001** |

n=9,693. SD, standard deviation. CES-D indicates Center for Epidemiologic Studies Depression Scale-8; LIBRA, LIfestyle for BRAin health. P-values are presented for the comparison of individuals with and without dementia at follow-up (independent samples t-tests, Mann-Whitney U-tests, and χ^2^-tests). LIBRA score theoretical range: –5.9 to 9.5; observed range: –5.9 to 9.5. ^*^ Only assessed in subsample.

**eTable 4.** Decomposed associations of depressive symptoms with dementia in total study population and subpopulation aged ≥70 years

|  | **B** | | **SE** | | **HR** | | | **Lower CI** | **Upper CI** | ***p*-value** |
| --- | --- | --- | --- | --- | --- | --- | --- | --- | --- | --- |
| **Total study population (****n=7,460; 306 dementia cases)** | | | |  | |  |  |  |  |  |
| Depressive symptoms on LIBRA | 1.10 | | 0.08 | | - | | | - | - | <0.001 |
| LIBRA direct on dementia | - | | - | | 1.09 | | | 1.05 | 1.14 | <0.001 |
| Depressive symptoms direct on dementia | - | | - | | 1.49 | | | 1.13 | 1.97 | 0.004 |
| Depressive symptoms indirect on dementia | - | | - | | 1.10 | | | 1.05 | 1.16 | <0.001 |
| Depressive symptoms total on dementia | - | | - | | 1.64 | | | 1.25 | 2.16 | <0.001 |
| **Age ≥70 years (n=2,445; 229 dementia cases)** | |  |  | |  | | |  |  |  |
| Depressive symptoms on LIBRA | 0.95 | | 0.13 | | - | | | - | - | <0.001 |
| LIBRA direct on dementia | - | | - | | 1.08 | | | 1.03 | 1.13 | 0.002 |
| Depressive symptoms direct on dementia | - | | - | | 1.11 | | | 0.80 | 1.54 | 0.541 |
| Depressive symptoms indirect on dementia | - | | - | | 1.07 | | | 1.02 | 1.12 | 0.004 |
| Depressive symptoms total on dementia | - | | - | | 1.19 | | | 0.86 | 1.64 | 0.297 |

LIBRA indicates LIfestyle for BRAin health; B, unstandardized regression coefficient; SE, standard error; HR, hazard ratio; CI, confidence interval. Analyses are adjusted for age, sex, educational level, wealth and clustering at the household level.

**eTable 5**. Interactions of depressive symptoms with age, sex, educational level, wealth and lifestyle on dementia

| **Interaction** | ***p-*value** |
| --- | --- |
| **Depressive symptoms on dementia** |  |
| Depressive symptoms *age | **0.002** |
| Depressive symptoms *women | 0.207 |
| Depressive symptoms *low education * | 0.147 |
| Depressive symptoms *medium education † | 0.208 |
| Depressive symptoms *high education † | 0.331 |
| Depressive symptoms *low wealth * | 0.628 |
| Depressive symptoms *medium wealth † | 0.958 |
| Depressive symptoms *high wealth † | 0.386 |
| Depressive symptoms *LIBRA | 0.592 |

n=7,460; 306 dementia cases. LIBRA indicates LIfestyle for BRAin health. * Compared to medium/high education level or wealth. † Compared to low education level or wealth. Analyses are adjusted for age, sex, educational level, wealth and clustering at the household level.

**eTable 6.** Associations of depressive symptoms with dementia in subpopulation aged 50-70 years

|  | **HR** | **Lower CI** | **Upper CI** | | ***p*-value** |
| --- | --- | --- | --- | --- | --- |
| Subpopulation ≥6 years of follow-up (n=4,215; 48 dementia cases) | 3.26 | 1.87 | | 5.69 | <0.001 |
| Additional adjustment for anti-depressant use and/or counseling in past 2 years (n=5,014; 73 dementia cases) | 3.13 | 1.93 | | 5.06 | <0.001 |
| Participants with 1 or 2 missing items on CES-D classified as missing (n=4,981; 73 dementia cases) | 3.16 | 1.98 | | 5.02 | <0.001 |

HR indicates hazard ratio; CI, confidence interval. Analyses are adjusted for age, sex, educational level, wealth and clustering at the household level.

**eTable 7.** Stratified analyses by sex, educational level and wealth

|  | **B** | | | **SE** | | | | **HR** | | | **Lower CI** | **Upper CI** | ***p*-value** |
| --- | --- | --- | --- | --- | --- | --- | --- | --- | --- | --- | --- | --- | --- |
| **Women (n=4,096; 181 dementia cases)** | | |  |  | | | |  | | |  |  |  |
| Depressive symptoms on LIBRA | **1.06** | | | **0.10** | | | | - | | | - | - | **<0.001** |
| LIBRA direct on dementia | - | | | - | | | | **1.10** | | | **1.05** | **1.16** | **<0.001** |
| Depressive symptoms direct on dementia | - | | | - | | | | 1.29 | | | 0.92 | 1.79 | 0.139 |
| Depressive symptoms indirect on dementia | - | | | - | | | | **1.11** | | | **1.05** | **1.17** | **<0.001** |
| Depressive symptoms total on dementia | - | | | - | | | | **1.43** | | | **1.03** | **1.98** | **0.033** |
| **Men (n=3,364; 125 dementia cases)** | |  | |  | | | |  | | |  |  |  |
| Depressive symptoms on LIBRA | **1.16** | | | **0.14** | | | | - | | | - | - | **<0.001** |
| LIBRA direct on dementia | - | | | - | | | | 1.07 | | | 0.99 | 1.15 | 0.074 |
| Depressive symptoms direct on dementia | - | | | - | | | | **1.94** | | | **1.26** | **2.98** | **0.003** |
| Depressive symptoms indirect on dementia | - | | | - | | | | 1.08 | | | 0.99 | 1.18 | 0.085 |
| Depressive symptoms total on dementia | - | | | - | | | | **2.09** | | | **1.34** | **3.26** | **0.001** |
| **Low educated (n=2,974; 161 dementia cases)** | | | | | |  |  |  | | |  |  |  |
| Depressive symptoms on LIBRA | **0.89** | | | **0.11** | | | | - | | | - | - | **<0.001** |
| LIBRA direct on dementia | - | | | - | | | | **1.08** | | | **1.03** | **1.15** | **0.004** |
| Depressive symptoms direct on dementia | - | | | - | | | | 1.25 | | | 0.87 | 1.78 | 0.228 |
| Depressive symptoms indirect on dementia | - | | | - | | | | **1.08** | | | **1.02** | **1.13** | **0.007** |
| Depressive symptoms total on dementia | - | | | - | | | | 1.34 | | | 0.94 | 1.92 | 0.111 |
| **Medium educated (n=2,028; 76 dementia cases)** | | | | | | | | |  |  |  |  |  |
| Depressive symptoms on LIBRA | **1.30** | | | **0.16** | | | | - | | | - | - | **<0.001** |
| LIBRA direct on dementia | - | | | - | | | | 1.07 | | | 0.98 | 1.17 | 0.122 |
| Depressive symptoms direct on dementia | - | | | - | | | | **2.15** | | | **1.26** | **3.66** | **0.005** |
| Depressive symptoms indirect on dementia | - | | | - | | | | 1.09 | | | 0.98 | 1.23 | 0.129 |
| Depressive symptoms total on dementia | - | | | - | | | | **2.35** | | | **1.37** | **4.04** | **0.002** |
| **High educated (n=2,454; 68 dementia cases)** | | | | | |  |  |  | | |  |  |  |
| Depressive symptoms on LIBRA | **1.04** | | | **0.16** | | | | - | | | - | - | **<0.001** |
| LIBRA direct on dementia | - | | | - | | | | **1.11** | | | **1.01** | **1.23** | **0.041** |
| Depressive symptoms direct on dementia | - | | | - | | | | 1.77 | | | 0.99 | 3.16 | 0.056 |
| Depressive symptoms indirect on dementia | - | | | - | | | | 1.12 | | | 1.00 | 1.25 | 0.059 |
| Depressive symptoms total on dementia | - | | | - | | | | **1.98** | | | **1.10** | **3.55** | **0.023** |
| **Low wealth (n=2,329; 120 dementia cases)** | | | | |  | |  |  | | |  |  |  |
| Depressive symptoms on LIBRA | **1.14** | | | **0.13** | | | | - | | | - | - | **<0.001** |
| LIBRA direct on dementia | - | | | - | | | | **1.09** | | | **1.02** | **1.16** | **0.008** |
| Depressive symptoms direct on dementia | - | | | - | | | | 1.43 | | | 0.95 | 2.14 | 0.090 |
| Depressive symptoms indirect on dementia | - | | | - | | | | **1.10** | | | **1.02** | **1.19** | **0.011** |
| Depressive symptoms total on dementia | - | | | - | | | | **1.57** | | | **1.04** | **2.36** | **0.031** |
| **Medium wealth (n=2,565; 107 dementia cases)** | | | | | |  |  |  | | |  |  |  |
| Depressive symptoms on LIBRA | **1.09** | | | **0.14** | | | | - | | | - | - | **<0.001** |
| LIBRA direct on dementia | - | | | - | | | | **1.09** | | | **1.01** | **1.18** | **0.032** |
| Depressive symptoms direct on dementia | - | | | - | | | | 1.40 | | | 0.85 | 2.30 | 0.184 |
| Depressive symptoms indirect on dementia | - | | | - | | | | **1.10** | | | **1.00** | **1.21** | **0.043** |
| Depressive symptoms total on dementia | - | | | - | | | | 1.54 | | | 0.92 | 2.58 | 0.099 |
| **High wealth (n=2,566; 79 dementia cases)** | | | | | |  |  |  | | |  |  |  |
| Depressive symptoms on LIBRA | **1.01** | | | **0.16** | | | | - | | | - | - | **<0.001** |
| LIBRA direct on dementia | - | | | - | | | | **1.10** | | | **1.01** | **1.20** | **0.030** |
| Depressive symptoms direct on dementia | - | | | - | | | | **1.76** | | | **1.01** | **3.06** | **0.046** |
| Depressive symptoms indirect on dementia | - | | | - | | | | **1.10** | | | **1.01** | **1.20** | **0.037** |
| Depressive symptoms total on dementia | - | | | - | | | | **1.94** | | | **1.12** | **3.34** | **0.018** |

LIBRA indicates LIfestyle for BRAin health; B, unstandardized regression coefficient; SE, standard error; HR, hazard ratio; CI, confidence interval. Analyses are adjusted for age, sex, educational level, wealth and clustering at the household level.

**eTable 8.** Characteristics of analytical study sample before and after imputation

|  | **Before (n=7,460)** | **After (n=8,933)** |
| --- | --- | --- |
| **Demographics** |  |  |
| Age, mean (SD) | 65.7 (9.4) | 66.1 (9.8) |
| Women, n (%) | 4,096 (54.9) | 4,928 (55.2) |
| Educational level, n (%)  Low  Medium  High | 2,974 (39.9)  2,028 (27.2)  2,454 (32.9) | 3,756 (42.1)  2,354 (26.4)  2,806 (31.5) |
| Wealth, n (%)  Low  Medium  High | 2,329 (31.2)  2,565 (34.4)  2,566 (34.4) | 2,926 (32.8)  3,048 (34.1)  2,959 (33.1) |
| **Depression** |  |  |
| Depression*, n (%) | 1,692 (22.7) | 2,130 (24.1) |
| **Health- and lifestyle factors** |  |  |
| Heart disease, n (%) | 751 (10.1) | 957 (10.7) |
| Diabetes, n (%) | 1,235 (16.6) | 2,160 (24.2) |
| Hypercholesterolemia, n (%) | 4,654 (62.4) | 5,073 (58.6) |
| Hypertension, n (%) | 4,484 (60.1) | 5,637 (64.3) |
| Obesity, n (%) | 2,421 (32.5) | 2,660 (32.7) |
| Smoking, n (%) | 975 (13.1) | 1,221 (13.7) |
| Low-to-moderate alcohol use, n (%) | 4,110 (55.1) | 4,564 (54.4) |
| Physical inactivity, n (%) | 2,007 (26.9) | 2,647 (29.6) |
| High cognitive and social activity, n (%) | 3,102 (41.6) | 3,400 (38.4) |
| Healthy diet, n (%) | 4,436 (59.5) | 5,085 (59.1) |
| LIBRA score, mean (SD) | 0.27 (3.02) | 0.54 (3.03) |
| **Dementia** |  |  |
| Incident dementia, n (%) | 306 (4.1) | 395 (4.4) |

LIBRA indicates LIfestyle for BRAin health, SD, standard deviation. P-values are presented for the comparison of individuals with and without dementia at follow-up (independent samples t-tests, Mann-Whitney U-tests, and χ^2^-tests). LIBRA score theoretical range: –5.9 to 9.5; observed range: –5.9 to 9.5. ^*^ Center for Epidemiologic Studies Depression Scale-8 score >=3 and self-reported diagnosis combined.

**References**

[1] World Health Organization. (2011). *Use of Glycated Haemoglobin (HbA1c) in the Diagnosis of Diabetes Mellitus: Abbreviated Report of a WHO Consultation.* World Health Organization, Geneva. <https://apps.who.int/iris/handle/10665/70523>

[2] National Health Service. High cholesterol. National Health Service, London. <https://www.nhs.uk/conditions/high-cholesterol/cholesterol-levels>. Accessed 07-11-2022.

[3] Guidelines Subcommittee. (1999). 1999 World Health Organization-International Society of Hypertension Guidelines for the Management of Hypertension. *Journal of Hypertension, 17*(2), 151-183. PMID: 10067786

[4] Radloff L.S. (1977). The CES-D Scale: A Self-Report Depression Scale for Research in the General Population. *Applied Psychological Measurement, 1*(3), 385-401. <https://doi.org/10.1177/014662167700100306>

[5] Turvey, C.L., Wallace, R.B., Herzog, R. (1999). A revised CES-D measure of depressive symptoms and a DSM-based measure of major depressive episodes in the elderly. *International Psychogeriatrics, 11*(2), 139-148. <https://doi.org/10.1017/s1041610299005694>

[6] World Health Organization (2008) *Waist circumference and waist–hip ratio: Report of a WHO expert consultation,* *Geneva, 8-11 December 2008*. World Health Organization, Geneva. <https://apps.who.int/iris/handle/10665/44583>

[7] Alcohol Policy Team, Department of Health (2016) *How to keep health risks from drinking alcohol to a low level Government response to the public consultation.* Department of Health. <https://assets.publishing.service.gov.uk/government/uploads/system/uploads/attachment_data/file/545911/GovResponse2.pdf>

[8] National Health Service. *5 A Day.* National Health Service, London. <https://www.nhs.uk/live-well/eat-well/5-a-day/portion-sizes>. Accessed 07-11-2022.
